# Editorial: Frozen elephant trunk surgery in aortic dissection

**DOI:** 10.3389/fcvm.2023.1154375

**Published:** 2023-03-10

**Authors:** Bleri Celmeta, Amer Harky, Antonio Miceli

**Affiliations:** ^1^IRCCS Ospedale Galeazzi - Sant'Ambrogio, Minimally Invasive Cardiac Surgery Unit, Milan, Italy; ^2^Department of Cardio-Thoracic Surgery, Liverpool Heart and Chest Hospital, Liverpool, United Kingdom

**Keywords:** frozen elephant trunk, FET, aortic arch, aortic dissection, TEVAR, hybrid, circulatory arrest, hypothermia

## Introduction

The aortic dissection (AD) is a potentially fatal disease, with up to 1% per hour death rate reported in the first several hours before surgery for type A dissection (1). The surgical treatment remains a highly complex procedure with a high risk of postoperative morbidity and mortality (2). However, in the past decades we have seen a progressive and steady evolution: until the 1980s the treatment involved the ascending aorta and aortic arch replacement followed by a thoraco-abdominal aortic replacement, with extremely high hospital and inter-procedure mortality. Subsequently, in 1983 the elephant trunk (ET) surgery was introduced by Borst et al. in order to facilitate the two-stage open technique for the surgical treatment of the descending aorta, avoiding the need of aortic cross-clamp (3). The introduction of endovascular technologies represented a fundamental turning point: the “conventional” trunk was replaced by a rigid stent graft in the mid-1990s to introduce a “one-step procedure” or to facilitate the realization of a two-stage hybrid treatment: this evolution gave birth to the frozen elephant trunk (FET) technique (4, 5). The techniques of cerebral perfusion and hypothermia have changed as well: we witnessed the evolution from deep hypothermic circulatory arrest, electrocerebral inactivity and no cerebral perfusion to mild hypothermia or normothermia and antegrade unilateral or bilateral selective cerebral perfusion: this contributed in reducing the rate of postoperative neurological and hypothermia-related complications (6–10). Today the evolution continues, not only by improving the techniques in cerebral, spinal and visceral protection but also by implementing the best advancements in minimally invasive and trans-catheter procedures: it is the case of partial sternotomy, hybrid (surgical and percutaneous) and completely trans-catheter replacement of the dissected aortic arch (11). This Topic and its collection of articles discuss some of the best advances in the field of FET surgery for various types of AD (Table 1).

**Table 1:** Summary of the collection of the articles.

**Authors**	**Title**	**Keywords**	**Summary**
Moula et al.	The evolution of arch surgery: Frozen elephant trunk or conventional elephant trunk?	• Frozen elephant trunk • Conventional elephant trunk • Evolution	In a metanalysis of 21 published articles between 2008 and 2021, ET was associated with higher early mortality but lower incidence of SCI compared to FET. No significant difference was found between ET and FET when only studies conducted in the last 5 years were included.
Luo et al.	Early and Long-Term Follow-Up for Chronic Type B and Type Non-A Non-B Aortic Dissection Using the Frozen Elephant Trunk Technique	• Type B aortic dissection • Type Non-A-Non-B aortic dissection • Follow-up	In 79 patients of Type B or Non-A-non-B AD operated from 2009 to 2019, the operation mortality rate was 5.1%. SCI occurred in 3.8% and stroke in 2.5%. At a median follow-up time of 53 months, overall survival rates were 96.2, 92.3, 88.0, 79.8, and 76.2% at 1/2, 1, 3, 5 and 7 years, respectively.
Fang et al.	Surgical Repair of Two Kinds of Type A Aortic Dissection After Thoracic Endovascular Aortic Repair	• Antegrade aortic dissection • Retrograde aortic dissection • TEVAR	The authors studied patients with AD post TEVAR which underwent FET surgery and divided them in two groups: retrograde AD (stent-induced AD) and antegrade AD (entry tear in the ascending aorta, unrelated with the distal stent). No significant differences in the incidence of post-operative complications or mortality were noted between the two groups.
Liu P. et al.	En Bloc Arch Reconstruction With the Frozen Elephant Trunk Technique for Acute Type A Aortic Dissection	• En bloc arch reconstruction • Island technique	In the authors' experience, with a hospital mortality rate of 9.8%, a stroke rate of 4.9% and a 3-year actuarial survival rate of 70.2%, the “island technique” appears to be feasible and effective with good early clinical follow-up results.
Wang et al.	Improvement of Clinical Outcomes of Total Aortic Arch Replacement and Frozen Elephant Trunk Surgery With Aortic Balloon Occlusion	• Aortic balloon occlusion • Circulatory arrest • Hypothermia	Aortic balloon occlusion technique can be safely performed in higher body temperatures (27.4^o^C) by significantly reducing circulatory arrest time (6.3 ± 5.7 min). In the authors' experience, it was associated with a lower 30-days mortality, fewer postoperative renal and hepatic injury, lower postoperative wake-up time, reduced chest tube output, lower red blood cell transfusion volume and no fatal events.
Tong et al.	Aortic Balloon Occlusion Technique Does Not Improve Peri-Operative Outcomes for Acute Type A Acute Aortic Dissection Patients With Lower Body Malperfusion	• Aortic balloon occlusion • Malperfusion	In patients presenting with acute type A AD and lower body malperfusion, the aortic balloon occlusion technique doesn't improve perioperative outcomes, which result similar to those of the conventional FET.
Liu Y. et al.	Efficacy of pump-controlled selective antegrade cerebral perfusion in total arch replacement: A propensity-matched analysis	• Selective antegrade cerebral perfusion • Pump-controlled cerebral perfusion • Single pump conjoint systemic and cerebral perfusion	The separate pump-controlled SACP may avoid cerebral flow inhomogeneity and potential cerebral injury during aortic arch surgery. However, when compared with a single pump flow for conjoint systemic and cerebral perfusion, no significant differences in perioperative neurological outcomes were found.
Liang et al.	Postoperative Hepatic Dysfunction After Frozen Elephant Trunk for Type A Aortic Dissection	• Hepatic dysfunction • Type A aortic dissection	Postoperative hepatic dysfunction after FET was associated with increased early mortality and morbidity, but not with late death in midterm follow-up.
Chivasso et al.	Systematic total arch replacement with thoraflex hybrid graft in acute type A aortic dissection: A single centre experience	• Postoperative outcomes • Type A aortic dissection • Frozen elephant trunk	The authors report a 30-days- and in-hospital mortality of 10.6 and 13.6%, respectively. The stroke and SCI rates were 4.5% and 3.0% respectively. LVEF, peripheral vascular disease, coronary malperfusion, lower limbs malperfusion, and CPB time were independent predictors of long-term mortality.
Wisniewski et al.	Single-Center Experience With the Thoraflex™ Hybrid Prosthesis: Indications, Implantation Technique and Results	• Postoperative outcomes • Frozen elephant trunk • Thoraflex™ Hybrid Prosthesis	The overall in-hospital mortality after FET for AD or aortic aneurysm was 12.5%. Permanent neurological dysfunction and SCI rates were 9.7 and 5.5%, respectively. At a mean follow-up of 26 ± 20 months, the mortality rate was 9.7%.
Tan, Jubouri et al.	What Is the Long-Term Clinical Efficacy of the Thoraflex™ Hybrid Prosthesis for Aortic Arch Repair?	• Long-term outcomes • Frozen elephant trunk • Thoraflex™ Hybrid Prosthesis	The authors describe the long-term outcomes of 931 cases of Thoraflex™ Hybrid implantation for aortic arch dissection, aneurysm, and penetrating atherosclerotic ulcer, finding that 30-day mortality was 0.6%, overall mortality was 1.5% while freedom from adverse events at 84 months was 95%.
Sheng et al.	Preoperative Nomogram and Risk Calculator for Postoperative Hypoxemia and Related Clinical Outcomes Following Stanford Type A Acute Aortic Dissection Surgery	• Nomogram • Risk calculator • Postoperative hypoxemia	Hypoxemia is frequent following acute AD surgery (24.2%) and is related to poorer clinical outcomes. Five independent risk factors for severe hypoxemia development were identified: older age, smoking history, renal insufficiency, higher body mass index, and white blood cell count.
Lin et al.	Prediction Nomogram for Postoperative 30-Day Mortality in Acute Type A Aortic Dissection Patients Receiving Total Aortic Arch Replacement With Frozen Elephant Trunk Technique	• Nomogram • Risk calculator • Postoperative 30-days mortality	LVEDD < 45 mm, GFR < 50 ml/min/1.73 m^2^, persistent abdominal pain, radiological celiac trunk malperfusion, concomitant CABG and CPB time >4 h were found to be independent predictors of the 30-day mortality in acute type A AD patients receiving total aortic arch replacement with FET technique.
Berger et al.	Distal Aortic Failure Following the Frozen Elephant Trunk Procedure for Aortic Dissection	• Distal aortic failure • Distal aortic reintervention • Distal stent graft-induced new entries • Frozen elephant trunk	Distal aortic failure assesses the treatment success of proximal aortic procedures with FET. The authors found that the incidence and risk for distal aortic failure following FET is very high (47.3%), especially in patients with more acute and more extensive aortic dissections or larger preoperative descending aortic diameters. They concluded that regular follow-up after FET is mandatory, since frequently FET is not a “single-step procedure”.
Jubouri et al.	Incidence of Distal Stent Graft Induced New Entry vs. Aortic Remodeling Associated With Frozen Elephant Trunk	• Distal stent graft-induced new entries • Aortic remodeling • Frozen elephant trunk	In this literature review, the authors conclude that (I) excessive oversizing of the stent-graft may induce dSINE; (II) a longer length of the stent-graft may promote false lumen thrombosis and aortic remodeling.
Walter et al.	Postoperative In-Stent Thrombus Formation Following Frozen Elephant Trunk Total Arch Repair	• Distal stent thrombosis • Frozen elephant trunk	In the authors' experience, distal stent thrombosis was a rare (6%), but highly relevant clinical event: distal embolization occurred in 21% of the patients with in-stent thrombosis, causing one in-hospital death by severe visceral ischemia. Female sex and aortic aneurysm were significant predictors for thrombus development. All patients received therapeutic anticoagulation, while overstenting with a stent-in-FET was the treatment in 11% of patients.
Kayali et al.	Kinking of Frozen Elephant Trunk Hybrid Prostheses: Incidence, Mechanism, and Management	• Distal stent kinking • Frozen elephant trunk	In this review, distal stent kinking after FET was found to be a rare (0-8%) but critical complication as it may result in intraluminal thrombosis and multi-organ embolism. The authors discuss the mechanism of the stent-graft kinking and the therapeutical and operative management of this life-threatening condition.
Yang et al.	Both J- and L-shaped upper hemisternotomy approaches are suitable for total arch replacement with frozen elephant trunk in patients with Type A dissection	• Minimally invasive • Upper hemisternotomy • Frozen elephant trunk	The authors retrospectively analyze their experience with FET performed by means of a minimally invasive access. They concluded that both J-shaped or L-shaped partial upper sternotomy are a feasible and safe option for FET surgery, in order to offer all the benefits of minimally invasive cardiac surgery.
Singh et al.	RELAY^TM^ Branched–International Results of Vessel Patency and Reintervention	• Percutaneous • Aortic arch TEVAR • RELAY^TM^ Branched endoprosthesis • Multicentric study	In this retrospective multicentric investigation of aortic arch TEVAR operation using the RELAY™ single-, double-, and triple-branched endoprostheses, technical success was achieved in 99.3% of cases. The 30-days mortality was 2.7% while the 30-days reintervention rate was 5.4%. Over a 24 months period, target vessel patency was maintained in 80.2% of patients, reintervention rate was 20.8% while no patient died.
Tan, Surkhi et al.	Does endovascular duration impact clinical outcomes in aortic arch repair? The RELAY™ branched international stance	• Percutaneous • Endovascular time • Aortic arch TEVAR • RELAY^TM^ Branched endoprosthesis	Longer endovascular operative duration of the RELAY™ branched endoprosthesis was associated with a lower likelihood of reintervention at 30 days, 6-, and 12 months and a greater likelihood of target vessel patency at 6- and 24 months. The prolonged endovascular duration may be the product of more extensive aortic arch repair.
Li et al.	Comparison of Prognosis Between Hybrid Debranching Surgery and Total Open Arch Replacement With Frozen Elephant Trunk for Type A Acute Aortic Syndrome Patients	• Debranching • Hybrid • Frozen elephant trunk	After a propensity score match analysis between patients having hybrid versus FET surgery, authors concluded that hybrid surgery could reduce aortic cross-clamp time and intraoperative hemorrhage. However, hybrid surgery was associated with increased incidence of permanent neurological complications, especially post-operative cerebral infarction.

FET: Frozen elephant trunk, ET: Elephant trunk, SCI: Spinal cord injury, AD: Aortic dissection, TEVAR: Thoracic endovascular aortic repair, SACP: Selective antegrade cerebral perfusion, LVEF: Left ventricular ejection fraction, CPB: Cardiopulmonary bypass, LVEDD: Left ventricular end-diastolic diameter, GFR: Glomerular filtration rate, CABG: Coronary artery bypass grafting, dSINE: Distal stent graft-induced new entries.

## Papers presentation

In their paper, Moula et al. conducted a metanalysis which aimed to investigate the differences in intrahospital outcomes of ET vs. FET. Twenty-one published articles between 2008 and 2021 with 3,153 patients were included. ET was associated with higher early mortality but lower incidence of SCI compared to FET. However, when studies published in the last 5 years were analyzed, no significant differences were found between ET and FET.

According to the position paper of Vascular domain of the EACTS, FET should be considered not only in type A AD with a primary entry in the distal aortic arch or in the proximal half of the descending aorta, but also in patients with complicated acute type B AD when primary TEVAR is not feasible (12). With this in mind, Luo et al. described their experience of early and long-term follow-up for chronic type B and type non-A non-B AD using the FET technique in 79 patients operated from 2009 to 2019. The operation mortality rate was 5.1%, SCI occurred in 3.8% and stroke in 2.5%. At a median follow-up time of 53 months, overall survival rates were 96.2, 92.3, 88.0, 79.8, and 76.2% at 1/2, 1, 3, 5, and 7 years, respectively. Fang et al. described their experience with FET in patients presenting with a type A AD following thoracic endovascular aortic repair (TEVAR). They divided the patients in 2 groups: the group of retrograde AD (stent-induced AD) and the one with an antegrade AD (entry tear in the ascending aorta, unrelated with the distal stent). All patients underwent total arch replacement and FET. No significant differences in the incidence of post-operative complications or mortality were noted between the two groups. In their paper, Liu P. et al. shared their experience with the en bloc arch reconstruction (island technique) with FET surgery for acute type A AD. With a hospital mortality rate of 9.8%, a stroke rate of 4.9% and a 3-year actuarial survival rate of 70.2%, they concluded that this technique appears to be feasible and effective with good early clinical follow-up results.

Aortic arch replacement generally contemplates a relatively long circulatory arrest (CA). This implies the use of cerebral perfusion/protection techniques during the CA on one side, and the establishment of moderate or deep hypothermia to protect all the other vital organs on the other side. Multiple postoperative complications are related, directly or indirectly, to hypothermia and CA: thrombocytopenia and deficiency of coagulation factors (thus causing a major risk of postoperative bleeding), alteration of the functions of the liver, kidneys, brain, pancreas, intestine and smooth muscles. Permanent neurological damage has been observed in 3 to 12% of patients when using circulatory arrest in deep hypothermia (13). Consequently, each effort to reduce CA and increase the core body temperature that permits a safely performed procedure is of outmost importance. To this purpose, Sénage et al. have described their technique: by placing two surgical sealing tourniquets around the aortic arch with the stent graft already deployed, the distal suture can be performed on a perfused aorta in normothermia and just a few minutes (4.5 ± 2.8 min) of CA (10). On the other hand, Wang et al. have described another way of dramatically reducing the CA: the aortic balloon occlusion (ABO) technique. Immediately after the stent-graft deployment, the sheathed aortic balloon is deployed inside the stent-graft. Once the balloon is fixed, lower body perfusion can be restarted through the femoral artery cannula, allowing a CA time of 6.3 ± 5.7 min and a mean nasopharyngeal temperature of 27.4°C. After propensity score matching of the ABO group and the conventional group, the first had significantly lower 30-days mortality, significantly fewer postoperative renal and hepatic injury, lower postoperative wake-up time, reduced chest tube output, lower red blood cell transfusion volume and no fatal events. In the particular subgroup of acute type A AD with lower body malperfusion, Tong et al. found no difference in perioperative outcomes between conventional and ABO in FET technique. According to the authors, the immediate true lumen perfusion in ABO technique may relive malperfusion only in dynamic obstruction, while it would have no effect on static malperfusion. This may explain to some extent the findings of the paper.

ASCP has allowed to gradually increase core body temperature during CA, but if it is carried out by using only one main arterial line, bifurcated with that of the systemic perfusion, brain flow is determined by peripheric and cerebral vascular resistance. This poses the risk of increased or inconstant cerebral flow which may result in neurologic injury. With this in mind, Liu Y. et al. shared their experience with a separate pump-controlled ASCP: even though no significant differences were found between separate pump and single pump flow, authors concluded that it is a safe and feasible procedure that may avoid cerebral flow inhomogeneity and potential cerebral injury.

Various papers have shared their insight on single-Center short and long-term results after FET. Liang et al. found that postoperative hepatic dysfunction after FET was associated with increased early mortality and morbidity, but not with late death in midterm follow-up. Chivasso et al. reported their single-Center experience with the FET technique: 30-days- and in-hospital mortality were 10.6 and 13.6%, respectively. Stroke occurred in 4.5% of patients. In total of 3.0% experienced spinal cord ischemia. Moreover, the authors found that left ventricular ejection fraction, peripheral vascular disease, coronary malperfusion, lower limbs malperfusion, and cardiopulmonary bypass time were independent predictors of long-term mortality. Wisniewski et al. evaluated the early and mid-term results after FET procedure for AD or aortic aneurysm in their Center, finding an overall in-hospital mortality of 12.5%, permanent neurological dysfunction and spinal cord injury (SCI) rates of 9.7 and 5.5%, respectively. At a mean follow-up of 26 ± 20 months, the mortality rate was 9.7%. Tan, Jubouri et al. have studied long-term outcomes of 931 cases of Thoraflex™ Hybrid implantation for aortic arch dissection, aneurysm, and penetrating atherosclerotic ulcer, finding that 30-day mortality was 0.6%, overall mortality was 1.5% while freedom from adverse events at 84 months was 95%.

Sheng et al. developed a preoperative nomogram and risk calculator for postoperative hypoxemia and related clinical outcomes after FET. Hypoxemia was frequent following acute AD surgery (24.2%) and was related to poorer clinical outcomes. Five independent risk factors for severe hypoxemia development were identified: older age, smoking history, renal insufficiency, higher body mass index, and white blood cell count. Lin et al. developed a nomogram model to predict postoperative 30-day mortality in acute type A AD patients receiving total aortic arch replacement with FET technique, by analyzing clinical data on 1,156 consecutive patients. Left ventricular end-diastolic diameter < 45 mm, estimated glomerular filtration rate < 50 ml/min/1.73 m^2^, persistent abdominal pain, radiological celiac trunk malperfusion, concomitant coronary artery bypass grafting and cardiopulmonary bypass time >4 h were found to be independent predictors of the 30-day mortality.

It has been demonstrated that the FET technique induces a precocious thrombosis of the false lumen in more than 90% of patients, followed by shrinkage and positive progressive remodeling over time (12, 14). This is why FET is frequently viewed as single stage procedure for pathologies involving the aortic arch. However, growing evidence is confirming that subsequent aortic reintervention after FET, regardless of the underlying aortic disease, is frequently needed. In order to assess the treatment success of proximal aortic procedures with FET, Berger et al. coined a new term: distal aortic failure. It was defined as: (I) distal aortic reintervention, (II) aortic diameter dilatation to ≥6 cm or growth of >5 mm within 6 months, (III) occurrence of a distal stent graft-induced new entries (dSINE) and (IV) aortic-related death. The authors found that the incidence and risk for distal aortic failure following the FET technique is very high (47.3%), especially in patients with more acute and more extensive aortic dissections or larger preoperative descending aortic diameters. The authors concluded therefore that regular follow-up after FET is mandatory. Jubouri et al. reviewed the incidence of dSINE and the rate of aortic remodeling after FET as a significant indicator of patient's prognosis. These two factors are closely interconnected, as the onset of a dSINE ensures a continuous perfusion of the false lumen and consequently a progressive increase of aortic diameter, impacting negatively on aortic remodeling. The authors concluded that (I) excessive oversizing of the stent-graft may induce dSINE; (II) a longer length of the stent-graft may promote false lumen thrombosis and aortic remodeling. However, other authors have expressed concerns that a longer stent-graft length may increase the risk of SCI due to a major cover of the spinal cord (15). More studies are needed to fully explore the correlation between dSINE, aortic remodeling, the length and graft size of the stent-graft. Walter et al. investigated the occurrence and clinical consequence of postoperative in-stent thrombosis following FET. In their experience, it was a rare (6%), but highly relevant clinical event: distal embolization occurred in 21% of the patients with in-stent thrombosis, causing one in-hospital death by severe visceral ischemia. Female sex and aortic aneurysm were significant predictors for thrombus development. All patients received therapeutic anticoagulation, while over stenting with a stent-in-FET was the treatment in 11% of patients. Kayali et al. reviewed the incidence and clinical consequences of stent-graft kinking after FET. It was found to be a rare (0–8% in the literature) but critical complication as it may result in intraluminal thrombosis and multi-organ embolism. In their paper, the authors discuss the mechanism of the stent-graft kinking and the therapeutical and operative management of this life-threatening condition.

Finally, several authors have discussed the implementation of the best advancements in minimally invasive, trans-catheter and hybrid procedures in aortic arch surgery. Yang et al. retrospectively analyzed their experience with FET performed by means of a minimally invasive access. They concluded that both J-shaped or L-shaped partial upper sternotomy are a feasible and safe option for FET surgery, in order to offer all the benefits of minimally invasive surgery. Singh et al. presented a retrospective European multicentric investigation of aortic arch TEVAR operation using the RELAY™ single-, double-, and triple-branched endoprostheses. Technical success was achieved in 99.3% of cases. The 30-days mortality was 2.7% while the 30-days reintervention rate was 5.4%. Over a 24 months period, target vessel patency was maintained in 80.2% of patients, while reintervention rate was 20.8%. No patient died during the follow-up. The authors concluded that the RELAY™ branched endoprosthesis show great promise as a new therapeutic aortic arch TEVAR for patients that may not be suitable for open surgical repair. Tan, Surkhi et al. found that longer endovascular operative duration of the RELAY™ branched endoprosthesis was associated with a lower likelihood of reintervention at 30 days, 6-, and 12 months and a greater likelihood of target vessel patency at 6- and 24 months. As the authors explained, this is likely an indirect correlation, as the prolonged endovascular duration may be the product of more extensive aortic arch repair. Li et al. studied the impact of a hybrid (traditional and trans-catheter) aortic arch treatment in acute type-A AD. Briefly, the authors firstly performed the open part with the reimplantation of the supra-aortic vessels to a prosthesis connected to the ascending aorta. A four-branch prosthetic graft was used whenever an ascending aorta replacement was needed. Next, the endovascular treatment part was performed and the stent-graft was released in the four-branched prosthetic graft or in the straight prosthetic graft. After a propensity score match analysis between patients having hybrid surgery vs. FET, authors found that hybrid surgery could reduce aortic cross-clamp time and intraoperative blood loss. However, hybrid surgery was associated with increased incidence of permanent neurological complications, especially post-operative cerebral infarction.

## Conclusion

In conclusion, this Topic collects some of the best and actuarial advancements in the field of the frozen elephant trunk surgery in aortic dissection. The evolution of the surgical treatment will surely pursue in order to further improve postoperative and follow-up outcomes in patients presenting with this potentially life-threatening condition.

## Author contributions

BC drafted the manuscript writing. All authors contributed substantially to the conception or design of the work. All authors contributed to the validation and final approval.
